# Investigating the Efficacy of Anatomical Silicone Models Developed from a 3D Printed Mold for Perineal Repair Suturing Simulation

**DOI:** 10.7759/cureus.3181

**Published:** 2018-08-22

**Authors:** Christine Goudie, Jessica Shanahan, Atamjit Gill, Deanna Murphy, Adam Dubrowski

**Affiliations:** 1 Med 3d Network, Memorial University of Newfoundland, St. John's, CAN; 2 Obstetrics and Gynecology, Memorial University of Newfoundland, St. John's, CAN; 3 Obstetrics and Gynecology, Memorial University, St. John's, CAN; 4 Emergency Medicine, Memorial University of Newfoundland, St. John's, CAN

**Keywords:** perineal repair, perineum, simulation, 3d printing, obstetrics, gynecology, postpartum, surgery

## Abstract

There is a scarcity of affordable, validated, standardized and anatomically correct silicone perineum models for the rehearsal of postpartum laceration repair. The purpose of this technical report is to describe and validate evidence for a silicone, perineal repair model created from a 3D printed mold for medical resident training and clinical skills maintenance.

A pre-existing model from an open-source royalty-free website was purchased and converted using Fusion360^TM^ (Autodesk Inc., San Rafael, CA, USA) into a stereolithography (.stl) file and altered to produce a negative mold. Using a spatula, a fine silicone layer was first applied inside the mold, followed by a small piece of flesh-colored mesh netting material within the perineal surface area, fitting the width of the mold. The mesh was pressed into the thin layer of silicone, which was meant to provide anatomical structure to prevent the sutures from tearing through the silicone. The remainder of the silicone mix was then poured into the mold, which required three hours to fully set before being removed from the mold. Twelve silicone models were produced and used during a one-hour workshop at the Rural and Remote Conference by 16 obstetrics and gynecology residents and practicing rural physicians, and four facilitators. At the end of the workshop, the participants were provided with a qualitative survey and asked to rate the perceived realism and educational effectiveness of the silicone perineum model as compared to pre-existing simulation models that they have used previously. The overall workshop participant feedback was positive, noting that the models provided more realistic visualization for the suturing simulation of first- and second-degree perineal injuries.

The silicone models were considered to be useful in simulation training when attempting first- and second-degreeperineum suturing techniques within a confined space. The overall feedback was positive, noting that they provided more realistic visualization experience compared to pre-existing simulation models, such as beef tongues and synthetic sponges. The feedback from the participants and facilitators included thoughts about how to add additional mesh to the silicone model so the subcutaneous and vaginal plane sutures would hold, as well as increase the size of the vaginal canal size to more accurately represent a postpartum repair. There were also suggestions to alter the colour of the model to be flesh-toned as opposed to pink, to more accurately simulate human tissue.

Silicone perineum models, created from a 3D printed mold, are an economical training tool as compared to commercially available, cost prohibitive models. They also provide anatomically accurate simulation training opportunities for residents to learn and maintain clinical skills in perineal repair, as compared to beef tongues and synthetic sponges, which have previously been used in obstetrics and gynecology simulation-based medical education.

## Introduction

Simulation‐based medical education (SBME) is rapidly advancing with tools such as three-dimensional printers to assist with the development of anatomically correct silicone models for the rehearsal of high acuity, low occurrence procedural skills [1]. Such models can be created from 3D printed molds, designed to provide more advanced haptic simulation for medical students and clinicians. Specialized fields such as obstetrics and gynecology benefit from such medical simulation opportunities to rehearse procedural skills in a safe learning environment, specifically to practice complex suturing patterns as applied in postpartum perineal repair [2]. Despite the emphasis on such procedural accuracy, a recent study revealed that a majority of obstetrics and gynecology students are not adequately prepared to independently perform such complex surgical skills at the end of their medical residency due to a lack of hands-on experience [3]. For simulation training purposes, trainees have commonly used synthetic sponges and raw beef tongues to represent female anatomy for the purpose of practicing perineal laceration suturing techniques [4]. However, such artificial objects and animal anatomy fail to accurately replicate the anatomical structure of female genitalia, thus does not provide ideal simulation training for such specialized procedures. Furthermore, the variability of animal anatomy does not allow for standardization of practice or consistent assessments [5]. Perineal injuries are a common occurrence during childbirth, affecting approximately 90% of women to varying degrees during natural vaginal delivery [6]. Perineal injuries involve the development of a laceration between the vaginal opening and anal sphincter region, also known as the perineum. Such injuries typically occur during prolonged labour, when the crown of the baby’s head is breaching the vaginal opening or when the shoulders pass, causing the perineum skin and or soft subcutaneous tissue to form a midline or mediolateral tear [7]. Perineal suturing occurs once the baby is delivered and the surgeon or resident can examine and categorize the severity of the soft tissue tear. The extent of the tear, determines its classification of severity, ranging from first and second degree, lower order magnitude wounds to third and fourth degree, higher order magnitude wounds [8]. Although the lower order lacerations are considered to be lower risk, higher frequency postpartum procedures, they can still present an increased risk to the patient if repaired incorrectly with complications including infection, chronic pain and sexual dysfunction [9]. The lower order lacerations are an area that family doctors are trained to repair; however, it is not a frequently rehearsed procedure due to the assistance of residents and more specialized obstetricians who often perform such procedures. The higher order, third and fourth-degree injuries, also known as obstetrical anal sphincter injuries (OASIS) are more complex in nature and can potentially cause fecal incontinence if repaired incorrectly, thus require more advanced suturing techniques and competency to repair [10].

The focus of this technical report is on the description of a novel, anatomical silicone perineum model, created from a 3D printed mold, to simulate the suturing of first- and second-degree perineal tears. Specifically, the mold was developed using the negative of a customized pre-existing stereolithography (.stl) file, which was printed, filled with silicone and the addition of a layer of mesh material for the purpose of holding sutures. This silicone perineum repair model served as a first iteration prototype for training and feedback purposes at the Rural and Remote Conference held in St. John’s on April 14, 2018.

## Technical report

**Figure 1:**
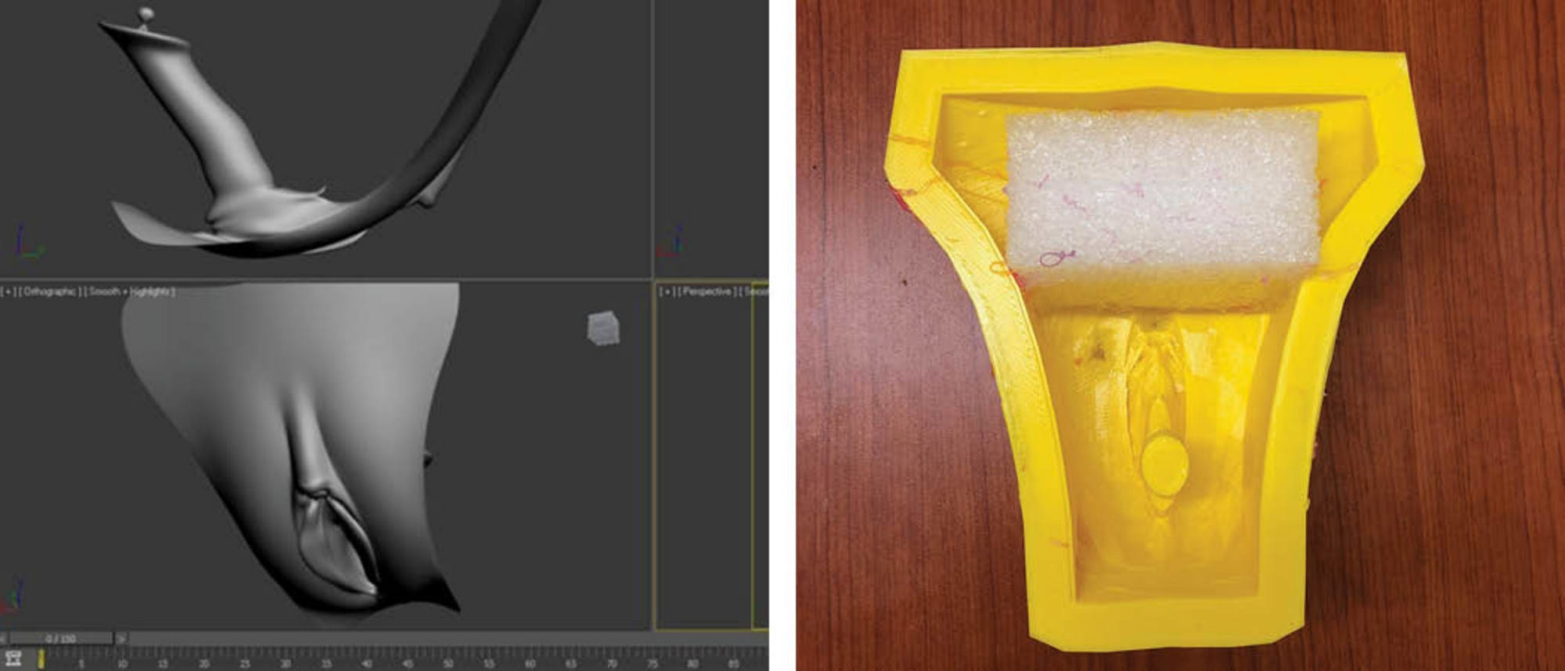
Perineum (.stl) model and custom 3D printed mold

A pre-existing model from an open source royalty free website (https://3dexport.com) was purchased and converted using Fusion360^TM^ (Autodesk Inc., San Rafael, CA, USA) into a stereolithography (.stl) file and altered to produce a negative mold (Figure 1) [11]. The 3D rendered mold in the form of a .stl file was transferred using a secure digital (SD) card, to an Ulimaker 2 3D printer and printed using polylactic acid (PLA) filament material. In total, 390g of PLA was used, taking the printer 10 hours and 56 minutes to complete the print. The file was printed using the Ulimaker 2 settings of 0.4 layer height with a 0.8 nozzle size. The 3D printed mold was altered post-printing to reduce unnecessary space at the top of the mold. A piece of ethafoam was used to reduce the mold space in preparation for the silicone fill (Figure 1).

**Figure 2:**
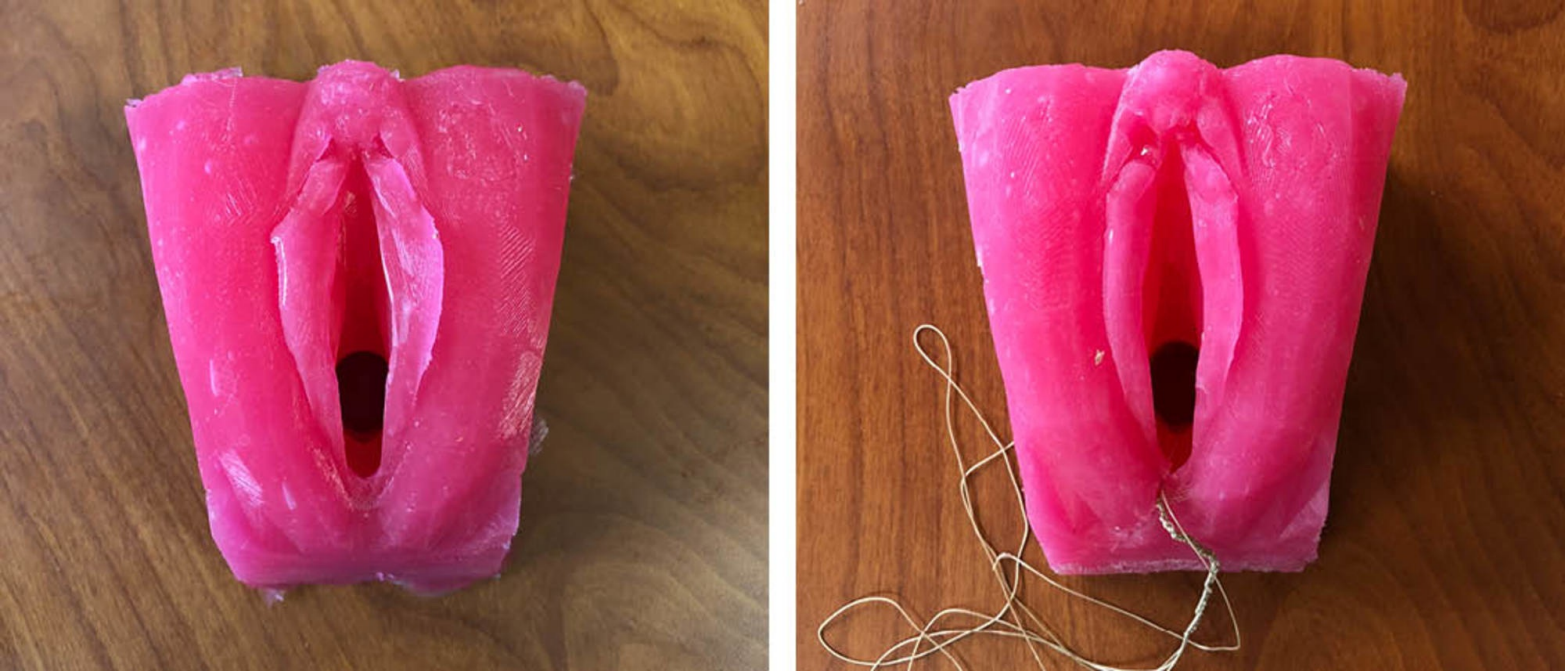
Silicone perineum model from 3D printed mold and silicone perineum model with sutured laceration from workshop

The silicone mix used was Smooth-On EcoFLex 00-30 Platinum Cure Silicone Rubber (Part A and Part B 1 = 1:1) measuring 200 ml in total using a large syringe. The silicone mix was combined with 1 tablespoon of Smooth-On Thi-Vex Silicone Thixotropic Agent (thickener). A small pea-sized amount of Smooth-On red silicone colouring was used, giving the models the bright pink colouring, which was an arbitrary colour choice. Using a spatula, a thin silicone layer was first applied inside the narrow end of the mold, followed by a small piece of flesh coloured mesh material, fitting the width of the mold. The mesh was pressed into the thin layer of silicone, which was meant to provide structure for the perineal plane as a way to prevent the sutures from tearing through the silicone. Each silicone mixture required three hours to fully set before being removed from the mold (Figure 2).

Context

**Figure 3:**
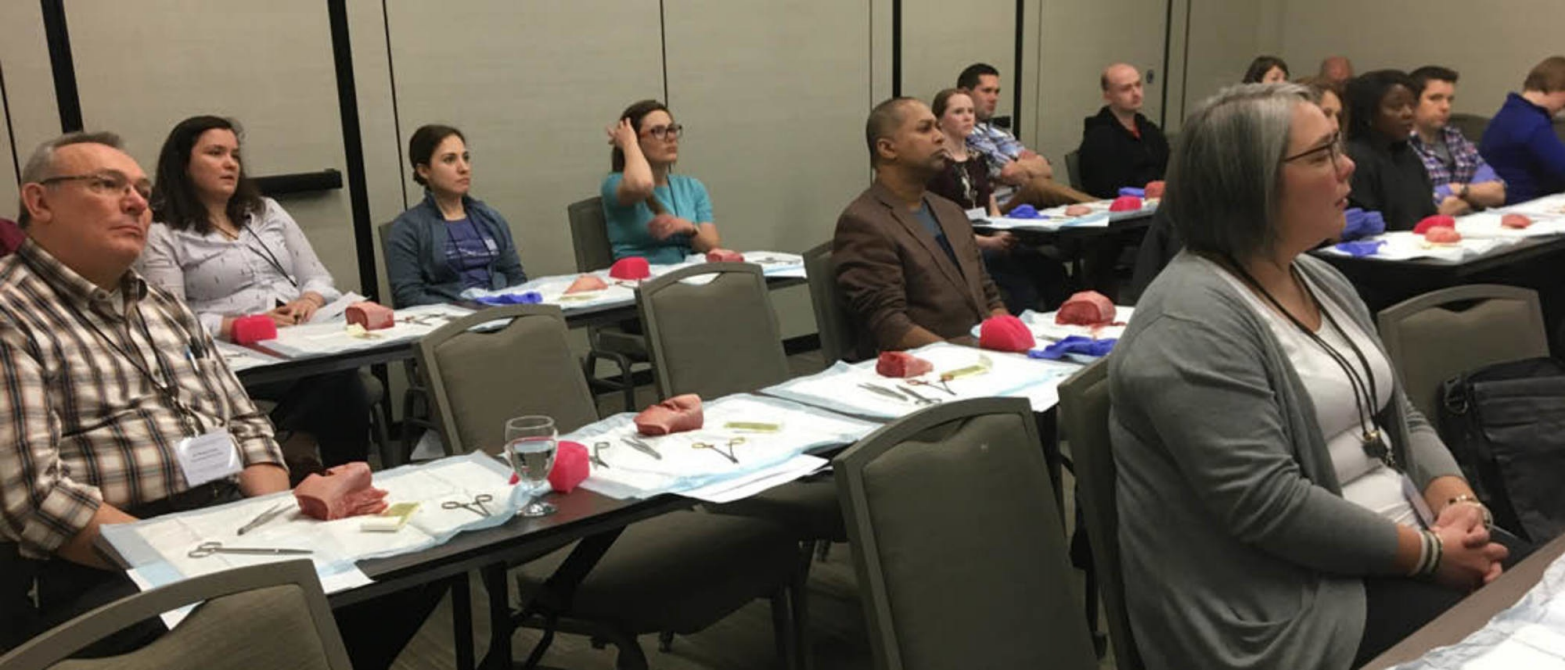
Obstetrics and gynecology perineum suturing workshop at the Remote and Rural Conference on April 14, 2018

The Rural and Remote Conference held in St. John’s on April 14, 2018, was held at the Delta Hotel in St. John’s, Newfoundland. The conference attracted rural health care practitioners, specifically, medical students, residents and family doctors from across Canada. Throughout the conference, various medical workshops were offered as a means to advance and maintain specialized skills required by rural family doctors and medical students. The perineal repair workshop had 16 participants (residents and practicing rural physicians) and was located within a breakout group lecture room, which contained six horizontal lecture tables, acting as the platform for which the participants performed the suturing upon (Figure 3).

The silicone perineum model served as a first iteration prototype for training and feedback purposes. The perineum suturing workshop was organized by the obstetrics and gynecology department at the Memorial University of Newfoundland (MUN) and was organized and hosted by Dr. Atamjit Gill and his obstetrics and gynecology residents Dr. Laura Williams, Dr. Annie Gu, and Dr. Jessica Shanahan. Dr. Atamjit Gill (M.B., B.S. India, FRCSC, FACOG) is an Associate Professor and Chair of Obstetrics at MUN. His residents Dr. Laura Williams, Dr. Annie Gu, and Dr. Jessica Shanahan are currently attending MUN for their obstetrics and gynecology residencies. The four facilitators provided workshop instructional context by showing a Powerpoint Presentation about perineum injuries, grades of severity and patterns for suturing repair.

Input

The equipment provided to residents included: toothed tissue forceps, suturing scissors, straight hemostat scissors, surgical absorbent pads, medical grade catgut suture and suturing needle, raw beef tongue, and silicone perineum model. The participants were presented with a Powerpoint slideshow of the suturing patterns to rehearse on each simulator. The four educators who circulated during the hands-on application segments included Dr. Gill and obstetrics and gynecology residents Dr. Laura Williams, Dr. Annie Gu, and Dr. Jessica Shanahan.

Process

**Figure 4:**
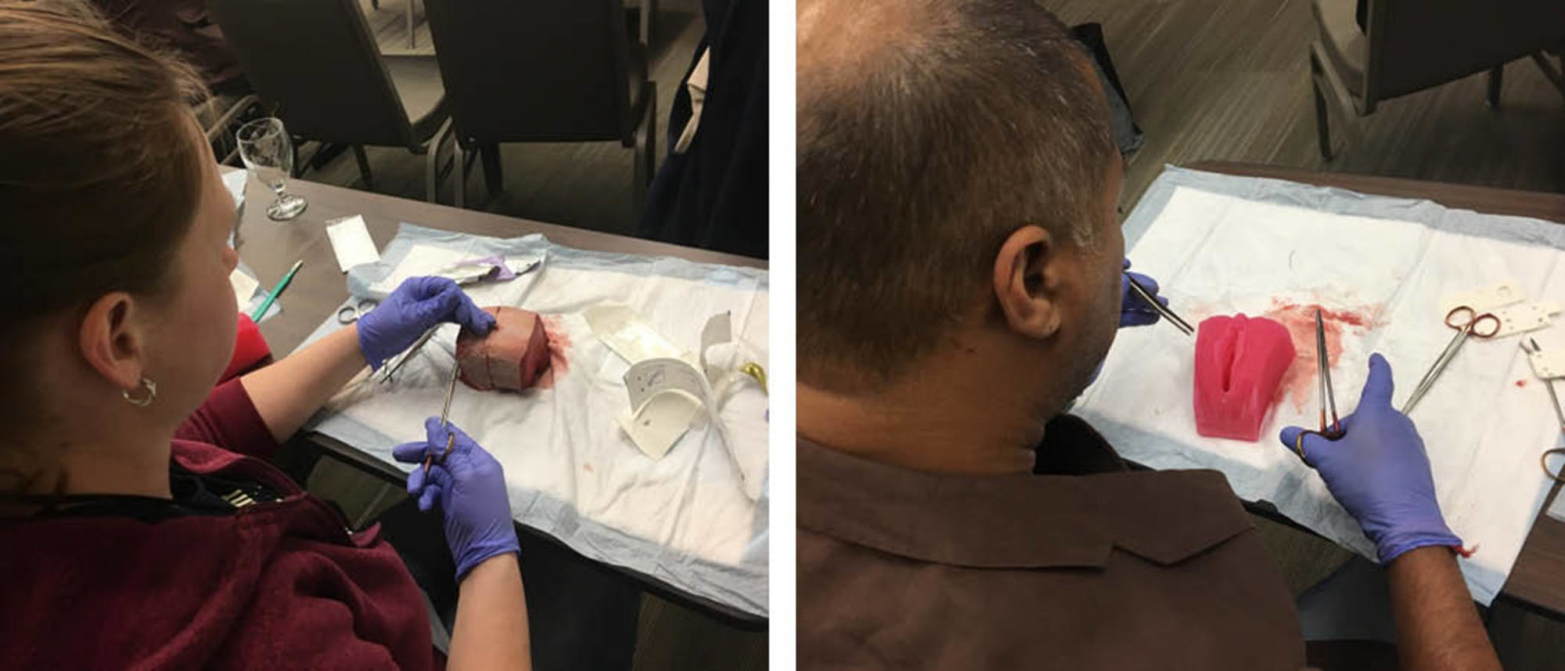
Beef tongue used for suturing simulation and silicone perineum model used for suturing simulation

Following the Powerpoint presentation slideshow, the participants were asked to apply the skills-based learning segment to their simulation models. No instruction was provided as to which model the participants were to begin with, however, it was reported that most participants voluntarily started with the beef tongue model to rehearse the suturing patterns, followed by the silicone model to rehearse the application of the suturing within a smaller, confined space (Figure 4).

Products/outcomes

**Figure 5:**
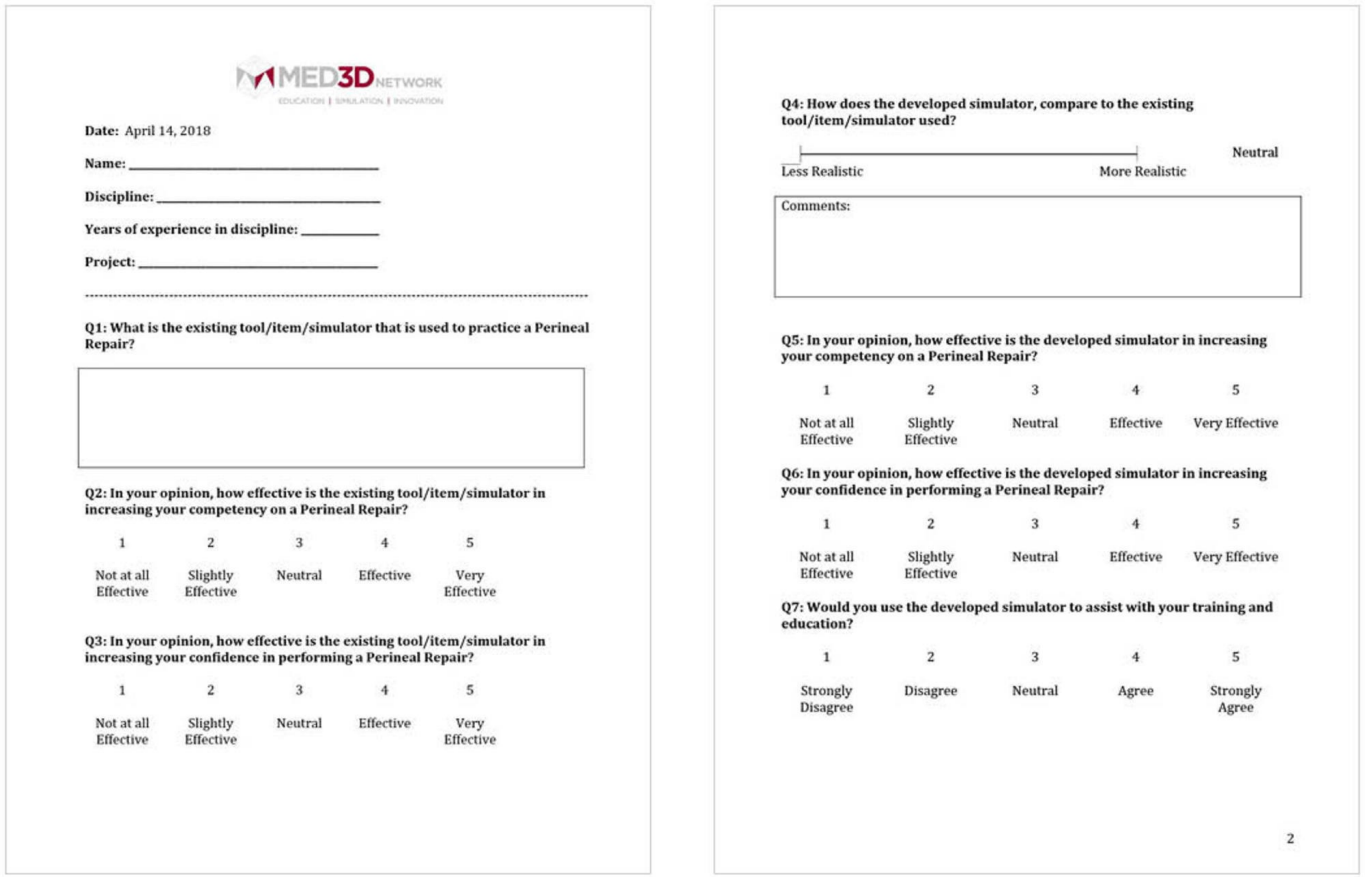
Efficacy survey to measure the performance of the silicone models compared to the pre-existing models

Twelve identical silicone perineum models were fabricated in total, which were altered by Dr. Jessica Shanahan, prior to the workshop, by making incisions mediolaterally to replicate a first or second-degree perineal lacerations. In addition to the silicone models, and upon receiving verbal consent from workshop participants, the facilitators provided a survey to determine the efficacy of the silicone model as a training tool as compared to the contrasting beef tongue models (Figure 5).

**Figure 6:**
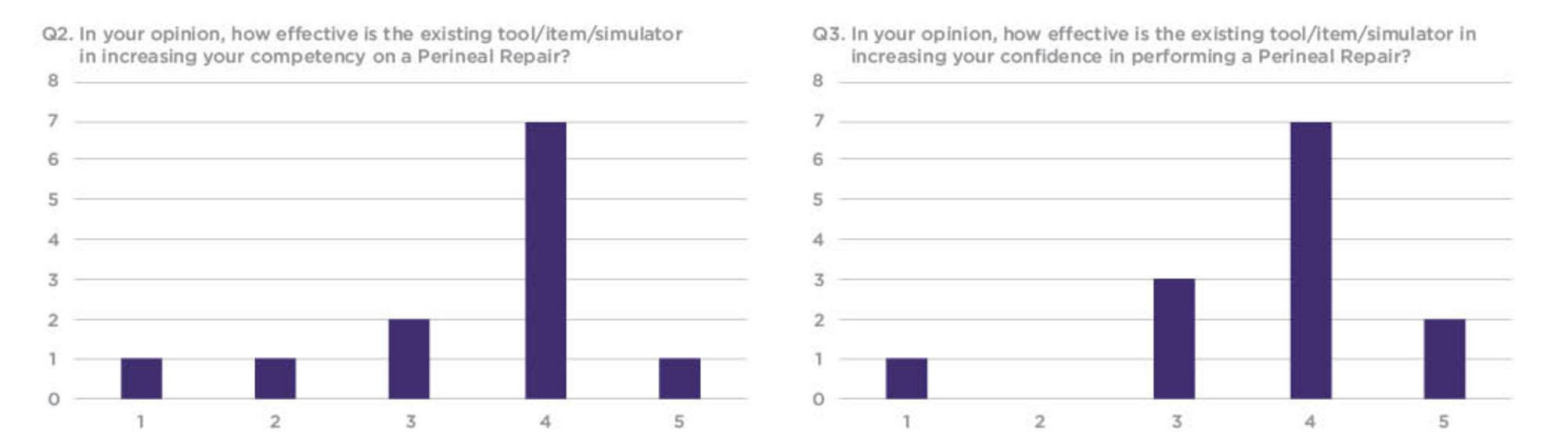
Q2 and Q3 results from the workshop participant feedback survey Y Axis: frequency of responses. X Axis: referring to the response anchors as related to each number respectively in Figure 5.

**Figure 7:**
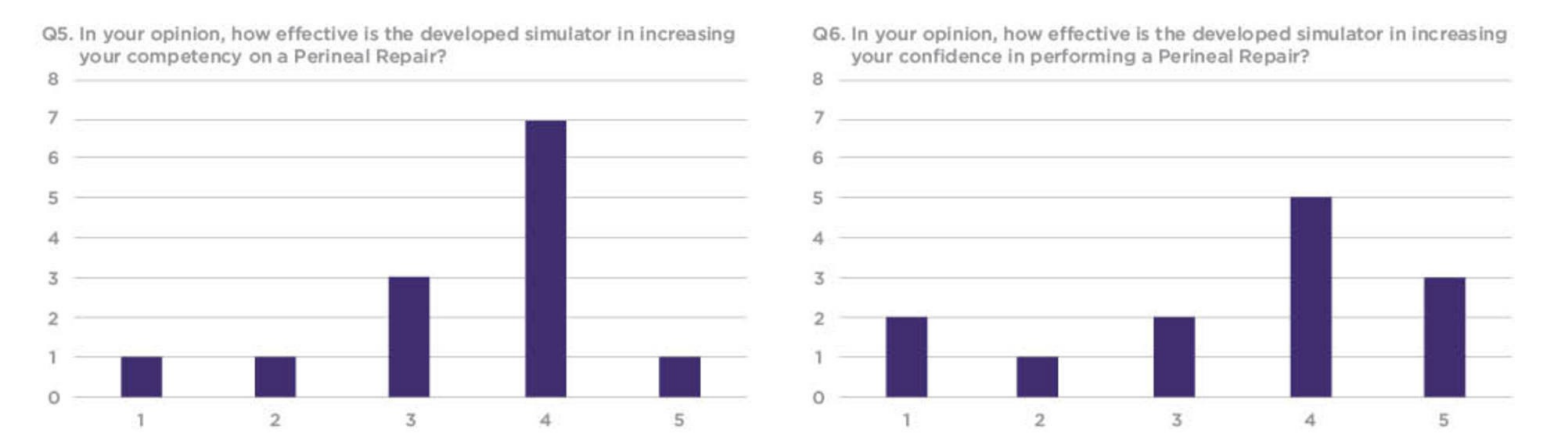
Q5 and Q6 results from the workshop participant feedback survey Y Axis: frequency of responses. X Axis: referring to the response anchors as related to each number respectively in Figure 5.

**Figure 8:**
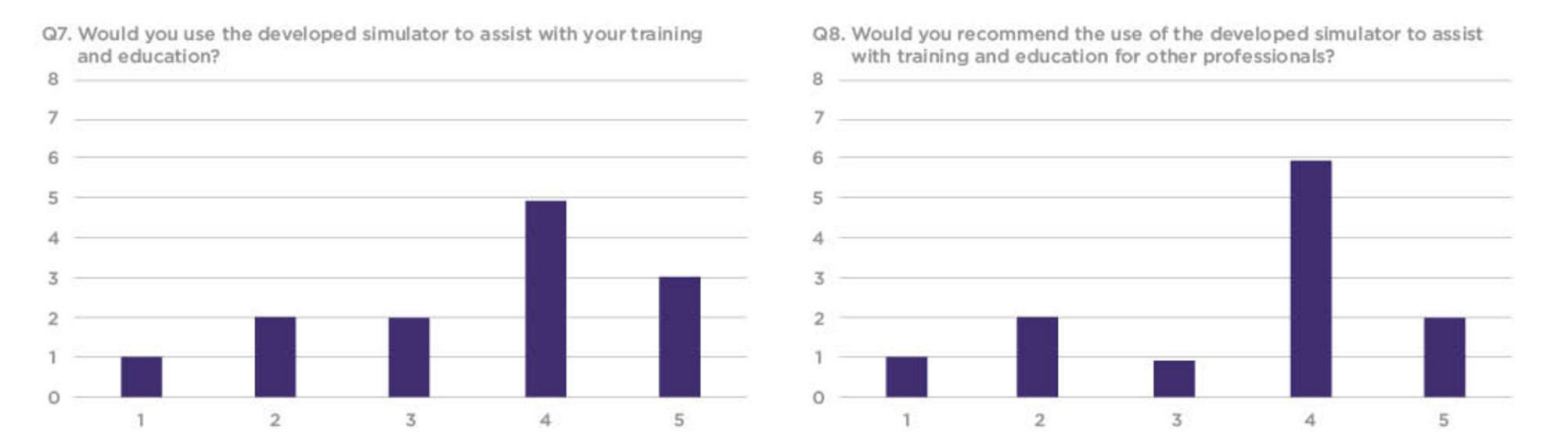
Q7 and Q8 results from the workshop participant feedback survey Y Axis: frequency of responses. X Axis: responses to questions 7 and 8. Referring to the response anchors as related to each number respectively in Figure 5.

The survey included 10 structured questions, with seven questions prompting participants to score between 1-5 on a linear Likert scale, one question prompting participants to rate the model on a sliding realism scale and two open-ended questions prompting feedback about the simulation training experience. Out of the 16 workshop participants who evaluated the model, 13 completed the distributed survey. Out of the 13, a total of 10 rated the model between 3-5 for simulation training efficacy and three rated the model between 1-2 (Figures 6-8).

## Discussion

**Figure 9:**
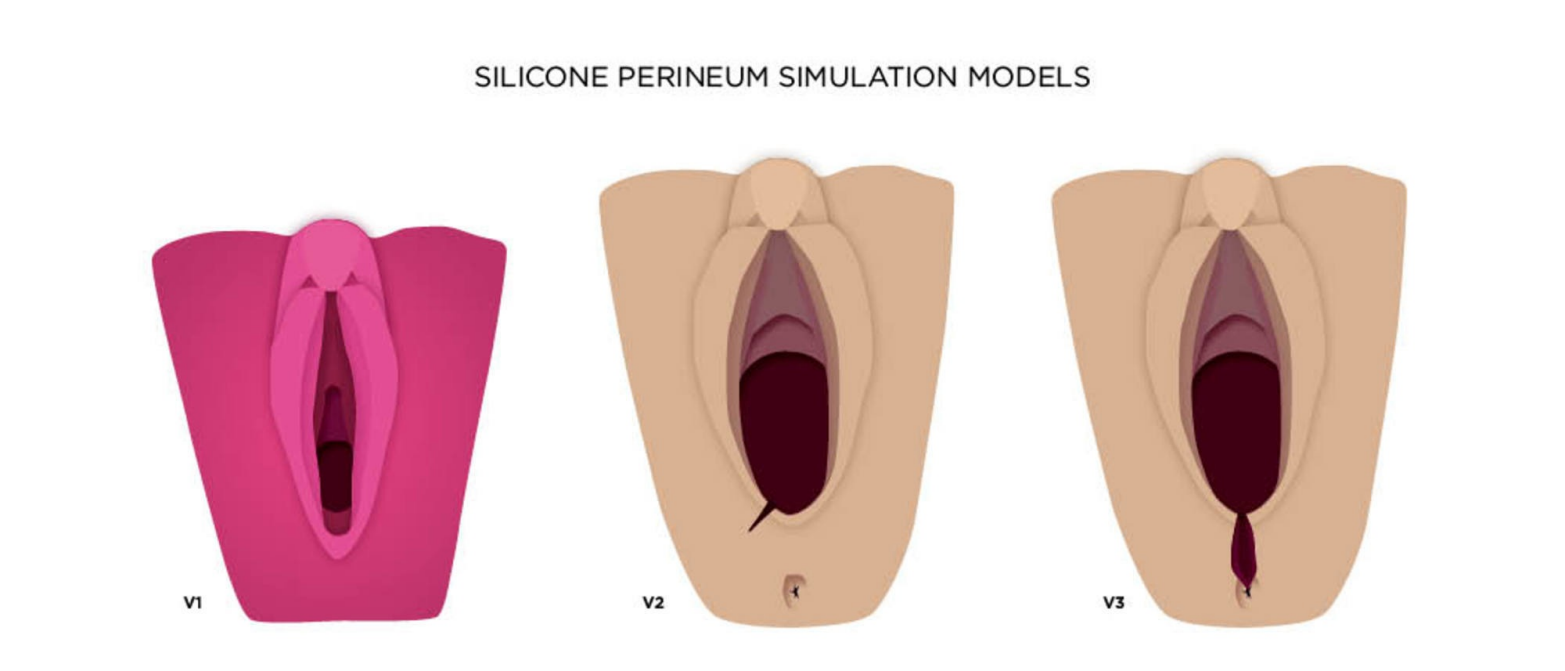
Proposed progression of silicone perineum models, which includes first- and second-degree lacerations (v2) and third- and fourth-degree lacerations (v3)

The silicone model was rated as useful in terms of providing an anatomically correct simulation tool for the suturing training. The response overall was quite positive and provided a number of changes required to make this a more accurate and fully functional simulation tool for future workshops and potentially before integration into an SBME learning curriculum. Specifically, feedback from the workshop facilitators as well as from Dr. Deanna Murphy, Assistant Professor of Obstetrics and Gynecology at the Memorial University of Newfoundland, included thoughts that the silicone models were slightly too small and narrow to be considered an accurate postpartum vaginal canal simulation model. Dr. Jessica Shanahan also suggested that the models should ideally include pre-made lacerations in the mold so the silicone tissue remains slightly ajar to more accurately represent the properties of soft tissue when torn. To provide a more accurate context for the simulation, ideally, the perineum models would also have blood and excess tissue surrounding the opening of the vaginal canal during the procedure. It was also noted that the sutures only held where the mesh netting was contained within the silicone and did not hold in the absence of the internal mesh. Consequently, future iterations should include mesh netting along the vaginal plane in addition to the current mesh along the perineal plane. There were also suggestions to replicate more accurate musculature layers near the perineal plane, as well as the addition of an anus, including muscular-type structure that would simulate that of a sphincter muscle. Finally, a 3D printed PLA stand should be integrated into the design to change the angle of positioning of the perineal model to 45 degrees, which would better simulate the position of the anatomy during such a perineal repair procedure. Dr. Gill also suggested that another model should be created, exclusively to rehearse third and fourth-degree lacerations (Figure 9).

**Figure 10:**
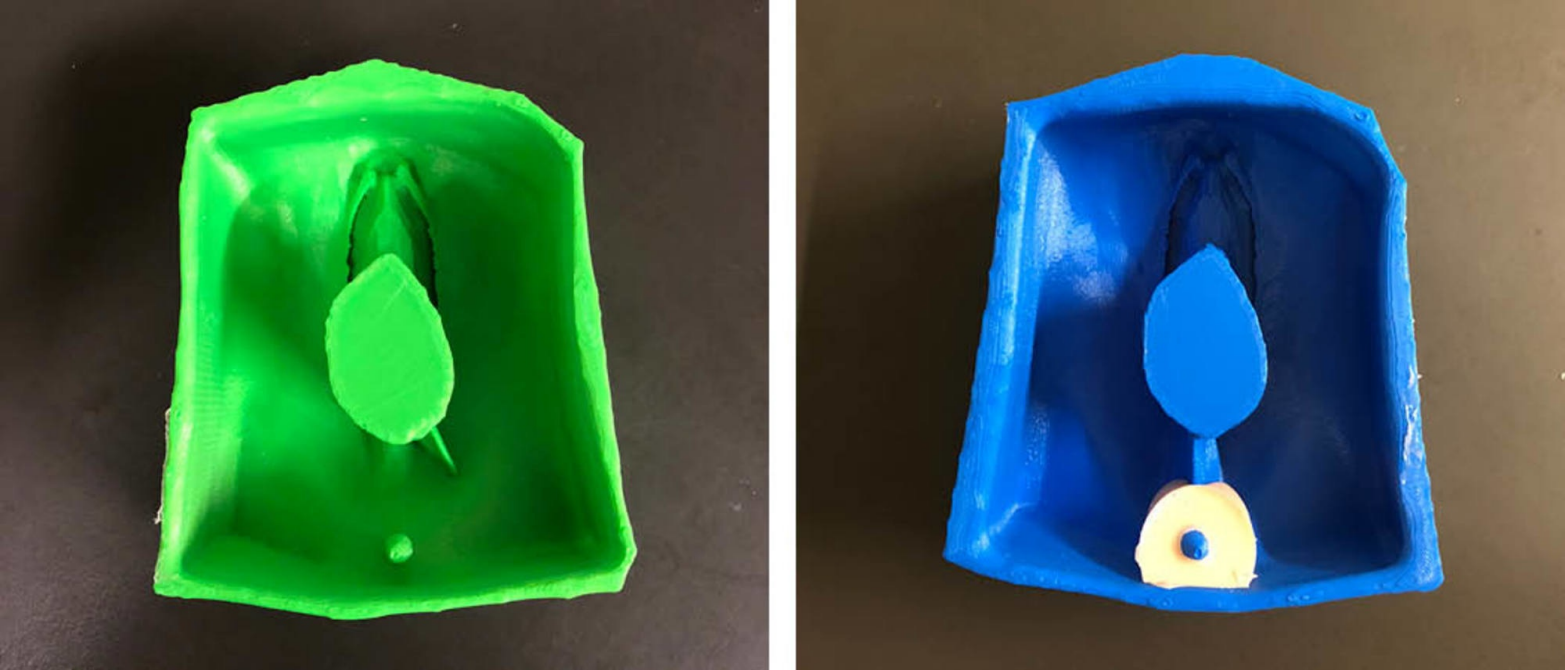
Second iteration molds for first- and second-degree perineal model (green) and third- and fourth-degree perineal model (blue)

Utilizing the workshop participant feedback in combination with the feedback from Dr. Jessica Shanahan, Dr. Atamjit Gill, and Dr. Deanna Murphy, a second iterative prototype mold was produced for both 1st and second-degree injuries as well as for third and fouth-degree repair (Figure 10).

**Figure 11:**
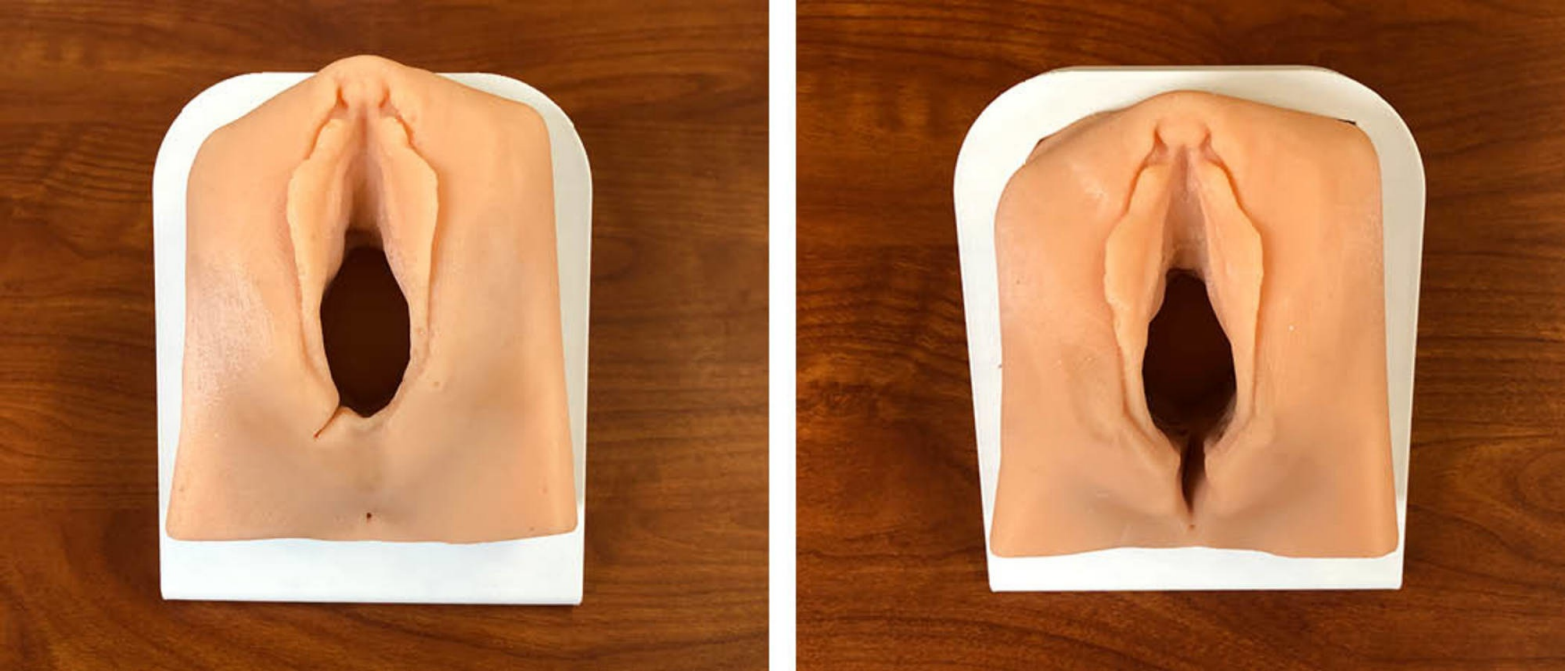
Second iteration of first- and second-degree perineal model and second iteration of third- and fourth-degree perineal model

The second iterative molds were used to produce the silicone models and included improvements such as flesh tone colour, strategic placement of mesh netting for suturing, pre-made lacerations, integration of an anus, wider vaginal canal, and a 3D printed base to tilt the models at a 45-degree angle (Figure 11). Currently, Dr. Atamjit Gill is planning to bring 25 of each revised perineum model to Bangladesh in September (2018) for the OBGYN Bangladesh International Alarm course as part of a Team Broken Earth outreach initiative, founded by Dr. Andrew Furey. The three-day workshop will focus on women's health and will provide an opportunity for clinicians to rehearse complex medical procedures such as caesarian sections and perineal laceration repair on silicone models. Workshop outcomes will be documented for a follow-up study involving the use of such models for simulation-based medical education in developing countries. 

## Conclusions

Silicone perineum models, created from 3D printed molds are a cost-effective method to more accurately teach and maintain perineal repair skills, compared to contrasting models such as beef tongues, currently used in obstetrics and gynecology SBME. The silicone models were thought to be beneficial because of the visual and physical realism. Specifically, they provide the constrained space and necessary anatomy where the learners can apply more fundamental suturing techniques. With suggested modifications made, the model can potentially be used at future obstetrics and gynecology conference workshops, within SBME, clinician skills maintenance, and for patient education.
